# Volatile molecules from bronchoalveolar lavage fluid can ‘rule-in’ *Pseudomonas aeruginosa* and ‘rule-out’ *Staphylococcus aureus* infections in cystic fibrosis patients

**DOI:** 10.1038/s41598-017-18491-8

**Published:** 2018-01-16

**Authors:** Mavra Nasir, Heather D. Bean, Agnieszka Smolinska, Christiaan A. Rees, Edith T. Zemanick, Jane E. Hill

**Affiliations:** 1 Geisel School of Medicine, Dartmouth College, 1 Rope Ferry Road, Hanover, NH 03755 United States; 2 School of Life Sciences, Arizona State University, 427 East Tyler Mall, Tempe, AZ 85287 United States; 3 NUTRIM School of Nutrition and Translational Research in Metabolism, Department of Pharmacology and Toxicology, Maastricht University, Maastricht, The Netherlands; 4 School of Medicine, Colorado Anschutz Medical Campus and Children’s Hospital Colorado, Department of Pediatrics, 13123 E 16th Avenue, Aurora, CO 80045 United States; 5 Thayer School of Engineering, Dartmouth College, 14 Engineering Drive, Hanover, NH 03755 United States

## Abstract

Respiratory infections caused by *Pseudomonas aeruginosa* and *Staphylococcus aureus* are the leading cause of morbidity and mortality in cystic fibrosis (CF) patients. The authors aimed to identify volatile biomarkers from bronchoalveolar lavage (BAL) samples that can guide breath biomarker development for pathogen identification. BAL samples (n = 154) from CF patients were analyzed using two-dimensional gas chromatography time-of-flight mass spectrometry. Random Forest was used to select suites of volatiles for identifying *P. aeruginosa*-positive and *S. aureus*-positive samples using multiple infection scenarios and validated using test sets. Using nine volatile molecules, we differentiated *P. aeruginosa*-positive (n = 7) from *P. aeruginosa*-negative (n = 53) samples with an area under the receiver operating characteristic curve (AUROC) of 0.86 (95% CI 0.71–1.00) and with positive and negative predictive values of 0.67 (95% CI 0.38–0.75) and 0.92 (95% CI 0.88–1.00), respectively. We were also able to discriminate *S. aureus*-positive (n = 15) from *S. aureus*-negative (n = 45) samples with an AUROC of 0.88 (95% CI 0.79-1.00) using eight volatiles and with positive and negative predictive values of 0.86 (95% CI 0.61–0.96) and 0.70 (95% CI 0.61–0.75), respectively. Prospective validation of identified biomarkers as screening tools in patient breath may lead to clinical application.

## Introduction

The leading cause of morbidity and mortality for patients with cystic fibrosis (CF) is respiratory failure associated with chronic bacterial infections and inflammation of the airways^1^. Prevalence of microorganisms varies with age, with *Staphylococcus aureus* and *Pseudomonas aeruginosa* being the most common bacterial species found in the airways of children and adults, respectively^2^. Accurate microbiological profiling of the lower airways and, consequently, prompt antibiotic treatment, are crucial for delaying chronic infections^3^. This is particularly true for *P. aeruginosa*, where eradication therapy is initiated whenever it is detected^4^. Early identification of *S. aureus*, both methicillin-sensitive (MSSA) and methicillin-resistant (MRSA), is also important, as MRSA is associated with increased risk of death, irrespective of CF disease severity^5^.

In expectorating patients, sputum is cultured for detection of bacterial pathogens. Most children as well as individuals with mild CF disease do not spontaneously expectorate sputum^6,7^. In non-expectorating patients, bronchoalveolar lavage (BAL) may be performed to diagnose lower airway infection. However, it is an invasive procedure that requires sedation and cannot be performed frequently. Hence, oropharyngeal (OP) swab cultures are used as surrogate specimens for species identification and antibiotic sensitivity determination of lower airway infections^8^. The current literature is divided on the utility of OP swabs as a reliable surrogate for BAL. Most studies on children report variable positive predictive values (PPV) ranging from 0.44 to 0.83 for *P. aeruginosa* and 0.33 to 0.64 for *S. aureus*^9–13^.

The measurement of volatile molecules from respiratory specimens, including BAL fluid, sputum and breath has been proposed as a minimally-invasive approach for differentiating *P. aeruginosa*-positive from *P. aeruginosa*-negative CF patients^14–22^. Diagnostic volatile molecular ‘suites’ from adult and pediatric patient breath have shown the greatest promise for detecting *P. aeruginosa*, with sensitivities and specificities approaching 1.00 and 0.88, respectively, in studies ranging from 16–233 subjects^17,23^. For *S. aureus* identification, Neerincx *et al*. showed that volatile molecules from patient breath (age ≥ 6 years) could be used to discriminate between *S. aureus*-infected CF patients (n = 13) from non-infected CF patients (n = 5) with a sensitivity of 1.00 and a specificity of 0.80^24^.

To set the groundwork for a breath study targeting biomarker evaluation of both *P. aeruginosa* and *S. aureus*, we accessed a heterogeneous set of *ex-vivo* BAL fluid samples (n = 154) obtained from 13 CF centers in the United States (US), via the Cystic Fibrosis Foundation Therapeutics (CFFT) biorepository. *P. aeruginosa* and *S. aureus*, the two most prevalent pathogens associated with CF, were detected by culture in 19% and 32% of samples, respectively. Validation sets were used to establish the sensitivity, specificity, PPV, and negative predictive value (NPV) for each set of putative volatile biomarkers.

## Materials and Methods

### Study subjects and design

BAL fluid samples stored at the Cystic Fibrosis Foundation Therapeutics (CFFT) Biorepository were accessed after approval from the Committee for the Protection of Human Subjects (CPHS) (STUDY00028597) at Dartmouth College. The stored samples were originally collected from subjects (age range: 2 months –50 years, n = 154) with a confirmed diagnosis of CF from 13 CF centers in the US at the time of clinically-indicated bronchoscopy with BAL fluid collection. Subjects with remnant BAL fluid after clinical testing were eligible to participate. The study was approved by the Institutional Review Board at each site. Written informed consent and HIPAA Authorization were obtained from all subjects ≥ 18 years or from parents or legal guardians of subjects < 18 years. Assent was obtained from subjects between 10–17 years. Clinical data (e.g., age, gender, lung function, body-mass index (BMI), and comorbidities) at the time of bronchoscopy were entered into a secure, web-based electronic database (REDCap).

### Specimen collection and processing

Bronchoscopy and BAL fluid collection were performed following each site’s standard clinical procedure with most samples collected via laryngeal mask airway or endotracheal tube, limiting upper airway contamination. Standard BAL fluid culture was performed by the local clinical microbiology laboratory in accordance with Cystic Fibrosis Foundation (CFF) guidelines^25^ and results recorded. Remnant BAL fluid was frozen neat in 1 mL aliquots within one hour of collection at −70 °C. Research samples collected at participating sites were batch-shipped overnight on dry ice to Children’s Hospital Colorado, United States for storage in the CFFT biorepository specimen bank. For volatile metabolomics analysis, cryovials were shipped overnight on dry ice to Dartmouth College, US and stored at −80 °C. Accompanying de-identified clinical and microbiologic data was shared electronically using encryption.

### Chemical measurements and data collection

Five hundred microliters of thawed BAL fluid were transferred to sterile 10 mL glass headspace vials containing a magnetic stir bar, and sealed with a polytetrafluoroethylene/silicone screw cap. Samples were stored at 4 °C prior to analysis by GC × GC-TOFMS. A total of 154 GC × GC chromatograms were analyzed, resulting in the detection of 973 volatile molecules across all samples (see Supplementary Information for details).

### Statistical analysis

The aim of the study was to investigate whether suites of volatile molecules discriminate between BAL fluid samples in each of the following comparisons: (1) *P. aeruginosa*-positive (*Pa*+) versus no cultured microorganism (NCM), (2) *S. aureus*-positive (*Sa*+) versus NCM, (3) culture-positive (*Pa*+/*Sa*+) versus NCM, (4) *Pa*+ versus *Sa*+, (5) *Pa*+ versus *P. aeruginosa*-negative (*Pa*−), and (6) *Sa*+ versus *S. aureus*-negative (*Sa−*). Clinical microbiology results obtained at the time of bronchoscopy were used as the gold standard. Samples labelled as NCM contained no reported pathogens but could be positive for respiratory flora. Descriptive statistics were calculated for age, gender, genotype, BMI, comorbidities (pancreatic insufficiency and cystic fibrosis-related diabetes (CFRD)), forced vital capacity (FVC) percent predicted, and forced expiratory volume in 1 s (FEV_1_) percent predicted. To assess confounding demographic variables between study groups, the Mann-Whitney U test^26^ with Benjamini-Hochberg (BH) correction^27^ was used to compare continuous variables (age, BMI, FEV_1_ percent predicted, FVC percent predicted) and Mantel-Haenszel chi-square test^28^ with BH correction was used to compare categorical variables (gender, genotype, pancreatic insufficiency, CFRD).

Preprocessing of chromatographic data decreases influence of artefacts and allows for biological variation to be visualized. Data were log-transformed and normalised using probabilistic quotient normalization^29^. Samples were randomly assigned to training sets for all six models. The Random Forest (RF) classification algorithm was performed on the training sets to identify suites of discriminatory volatile biomarkers^30^. RF creates many de-correlated decision trees from a randomly selected subset of volatile compounds and predicts the sample class assignment. Two-thirds of the samples in the training sets were randomly selected with replacement for each decision tree (with an equal number of samples selected per class) and the remaining one-third were used to calculate the performance of the RF classification model. A total of 1000 trees were built for each round of RF. Discriminatory volatile molecules were ranked based on their mean decrease in accuracy over a 100 independent rounds of RF. Volatile molecules that contributed to decrease in accuracy of >30% in at least 90/100 rounds were chosen as the most discriminatory features. A test set was used to validate the accuracy of selected discriminatory panel of volatile molecules for all models.

For visualization purposes, principal component analysis (PCA) score plot was used to demonstrate the relatedness between all samples in the data. In a PCA score plot, each single point is represented by a sample. Points that lie close to each other have similar volatile molecule profiles, while points that are distant have different properties. ROC curves were plotted to represent performance of the predictive models made by RF. The area under the ROC curve (AUROC) is used to assess predictive performance: a value close to 1 indicates high predictive power of the model, whereas an AUC close to 0.5 means that the model has no predictive power^31^. Confidence intervals (CIs) for sensitivity and specificity are exact Clopper-Pearson CIs. Confidence intervals for PPV and NPV, were calculated using standard logit CIs. CIs for AUROC were calculated using DeLong method. All statistical analyses were performed in R 3.3.2.

Multivariate analysis of variance (MANOVA) was performed to test the influence of confounders on the profiles of putative discriminatory volatile biomarkers. Of the 38 volatiles identified from all models, the Mann-Whitney U-test with BH correction was used to assess differences in the mean concentration between sample classes.

### Putative identification for volatile molecules

In total, 553, 400, and 200 chromatographic peaks were identified in at least one chromatogram of *Pa*+, *Sa*+ and NCM, respectively. Putative chemical class and chemical identification for volatile molecules that were present in at least 80% of one study group was based on mass spectral similarity score to a compound in the NIST library of at least 850/1000 and having a molecular weight of at least 60.0 amu. Volatile molecules that passed the above criteria and have been reported in the literature were assigned putative names. Experimentally-determined retention indices (RI) that are consistent with the volatile molecules assigned putative names on the mid-polar Rxi-624Sil stationary phase, were quantified using published median RIs for non-polar and polar columns, and the following equation:

$$\frac{{R}{{I}}_{{experimental}}-{R}{{I}}_{\text{non}{-}\text{polar}}}{{R}{{I}}_{{polar}}-{R}{{I}}_{\text{non}{-}\text{polar}}}\times 100 \% =5-35 \% $$

Retention indices less than 600 (corresponding to C_6_) or greater than 1600 (corresponding to C_16_) were not extrapolated.

### Pathway identification

For each putative volatile molecule assigned a name, the Kyoto Encyclopedia of Genes and Genomes (KEGG) database was searched for pathways identified in bacteria^32^.

### Data availability

The datasets generated during and/or analysed in the current study are available from the corresponding author on reasonable request.

## Results

### BAL fluid samples from subjects with cystic fibrosis are polymicrobial and heterogeneous

**Figure 1:**
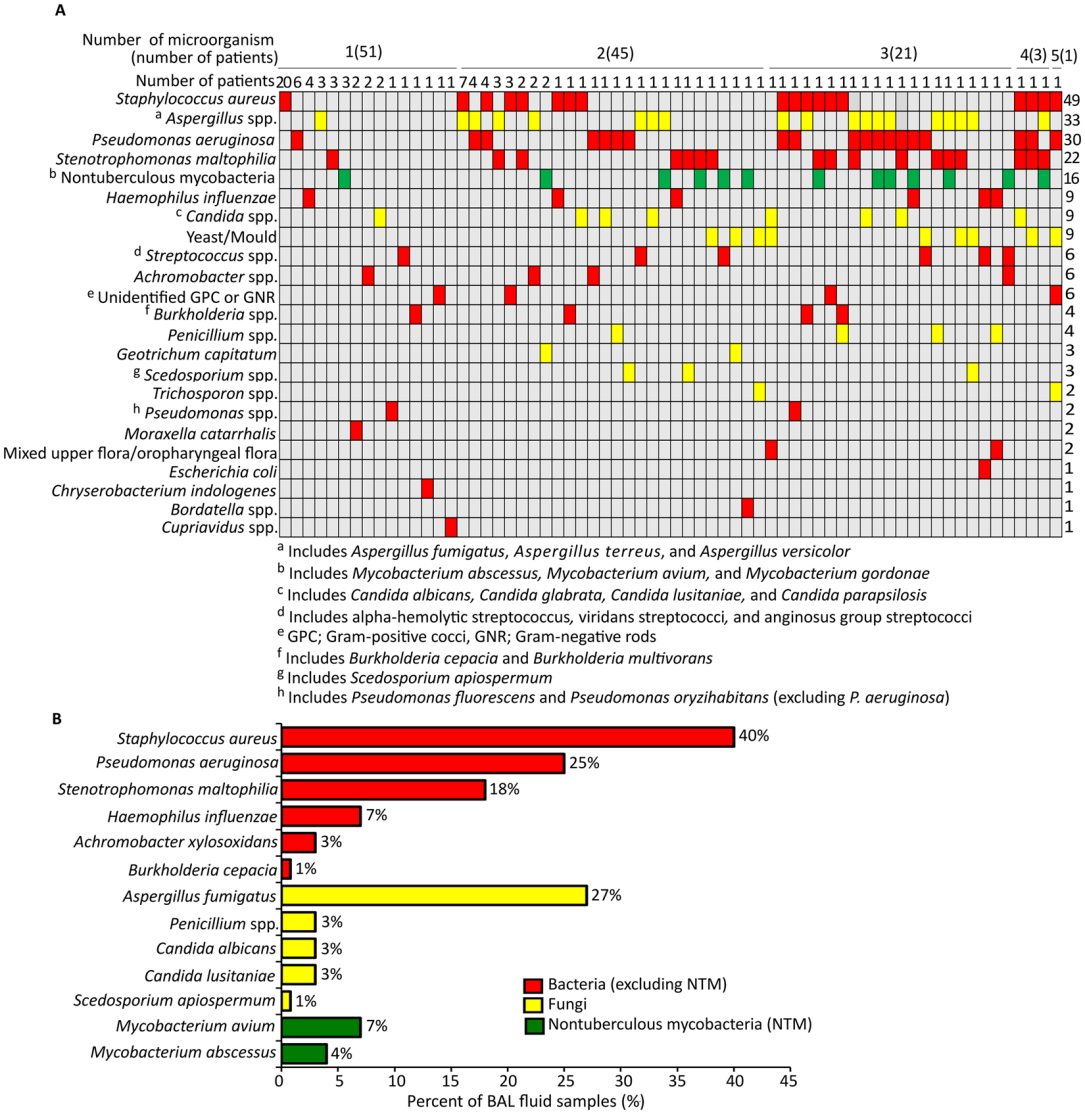
Microbiological results from BAL fluid samples of patients with cystic fibrosis (n = 121; samples with no cultured microorganisms (NCM) excluded for clarity). (**A**) Colour matrix of the microbiological profiles observed among the samples; (red) bacteria (excluding nontuberculous mycobacteria (NTM)), (yellow) fungi, (green) NTM. (**B**) Bar plot of the most prevalent microorganisms in the samples (for organisms present in ≥1% samples).

BAL fluid samples from subjects with CF (n = 154) were cultured for the identification of bacterial and fungal pathogens; 79% (n = 121) were identified as culture-positive and the remaining 21% (n = 33) culture-negative. The largest proportion of samples had only one culturable microorganism (n = 51), while 45, 21, 3, and 1 BAL fluid samples cultured two, three, four, and five microorganisms, respectively (Fig. 1A). Of the 66 unique microbiological profiles identified, 74% (n = 49) were specific to a single patient. Organisms cultured from at least 20% of BAL fluid samples were *S. aureus* (40%), followed by *Aspergillus fumigatus* (27%) and *P. aeruginosa* (25%) (Fig. 1B).

*S. aureus* and *P. aeruginosa* represent the two most prevalent pathogens in the setting of CF, therefore BAL fluid samples that cultured either *S. aureus* (*Sa*+) or *P. aeruginosa* (*Pa*+) were selected for further analysis. BAL fluid samples containing no cultured microorganism (NCM), *P. aeruginosa*-negative (*Pa−*) and *S. aureu*s-negative (*Sa*−) were used as comparison groups. For statistical analysis, we excluded data from samples that cultured both *S. aureus* and *P. aeruginosa* (n = 9) as well as 19 samples with incomplete clinical or demographic information. Age and BMI were significantly different in our *Pa*+ versus NCM and *Pa*+ versus *Pa*− model and could therefore confound our analysis (Table 1). We did not include medication in our analysis because the combination of drugs used by patients in our study groups exceeds the number of subjects in each group.

**Table 1:** Demographic and clinical characteristics of subjects in the study groups.

	Study groups^¥^					p-value					
	*Pa*+	*Sa*+	NCM	*Pa−*	*Sa*−	*P*+ vs NCM	*Sa*+ vs NCM	*Pa*+/*Sa*+vs NCM	*Pa*+ vs *Sa*+	*Pa*+ vs *Pa*−	*Sa*+ vs *Sa*−
n	19	40	32	114	93	—	—	—	—	—	—
Age	17.6 (±8.6)	13.3 (±5.2)	9.8 (±6.9)	10.5 (±6.2)	11.4 (±7.5)	*0.03	0.74	0.25	0.12	**0.003	0.72
Gender (M/F)	9/10	25/15	15/17	55/59	39/54	1.00	0.74	0.51	0.66	1.00	0.19
Genotype (*F508del*/*F508del */ *F508del*/*other)*	6/7	15/14	13/7	66/41	53/34	0.64	0.82	0.51	1.00	0.60	0.72
BMI, kg/m^2^	20.0 (±2.8)	18.2 (±3.7)	17.6 (±2.7)	17.9 (±3.2)	18.2 (±2.9)	*0.03	0.82	0.25	0.12	**0.01	0.94
Comorbidities (Y/N) Pancreatic insufficiency CF-related diabetes	17/2	33/7	21/11	110/4	90/3	0.19	0.74	0.25	0.95	0.60	0.10
	4/15	6/34	7/25	18/96	14/79	1.00	0.82	0.77	0.95	0.93	1.00
FVC % predicted Age ≥6 years	82.0 (±16.7) n = 18	94.6 (±15.6) n = 35	94.2 (±19.7) n = 22	91.5 (±18.8) n = 87	88.5 (±20.1) n = 69	0.07	0.82	0.51	0.12	0.08	0.72
FEV1% predicted Age ≥6 years	74.1 (±20.5) n = 18	87.1 (±17.9) n = 35	85.5 (±22.3) n = 22	83.4 (±20.7) n = 87	78.4 (±21.7) n = 69	0.19	0.82	0.50	0.13	0.16	0.22

^¥^Samples can belong to more than one study group.

Data shown as mean (standard deviation) except where indicated.

p-value <0.05*, 0.01** considered statistically significant after Benjamini-Hochberg correction.

M, male; F, female, Y, yes; N, no; FVC, forced vital capacity; FEV_1_, forced expiratory volume in 1 s.

### Volatile molecular profiles of study groups

**Figure 2:**
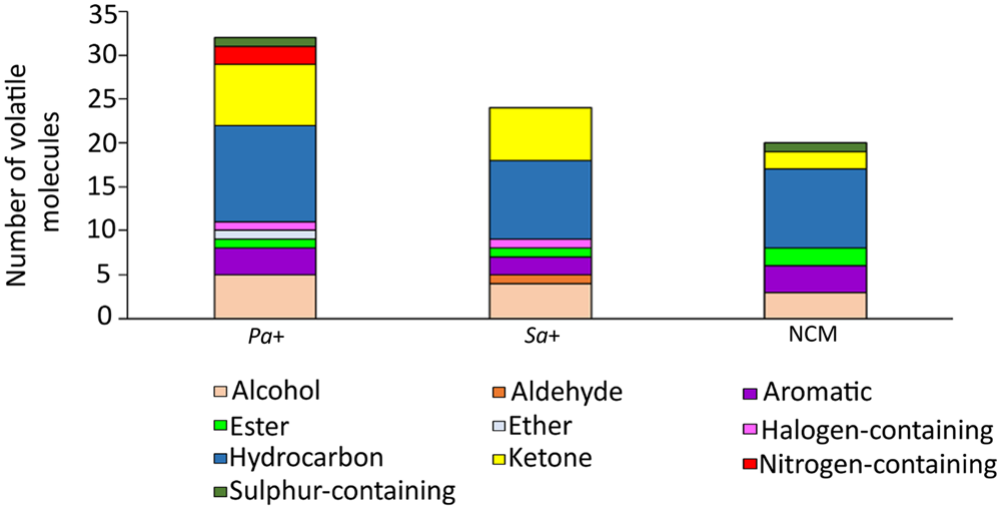
Putative chemical class identifications of volatile molecules present in BAL fluid samples of *Pa*+, *Sa*+, and NCM groups.

In total, 973 peaks were identified in the headspace of the BAL fluid samples (Supplementary Figure S1). After removal of chromatographic artefacts and known contaminants (e.g., siloxanes, phthalates, atmospheric gasses), 805 peaks were selected for further analysis and putative chemical class identifications were assigned to 60 molecules covering a variety of chemical classes. Hydrocarbons were the most abundant chemical class in each group (*Pa*+ = 11, *Sa*+ = 9, NCM = 9), followed by ketones (*Pa*+ = 7, *Sa*+ = 6, NCM = 2) and alcohols (*Pa*+ = 5, *Sa*+ = 4, NCM = 3) (Fig. 2).

### Identifying *P. aeruginosa* and *S. aureus* from BAL fluid samples using suites of volatile biomarkers

**Figure 3:**
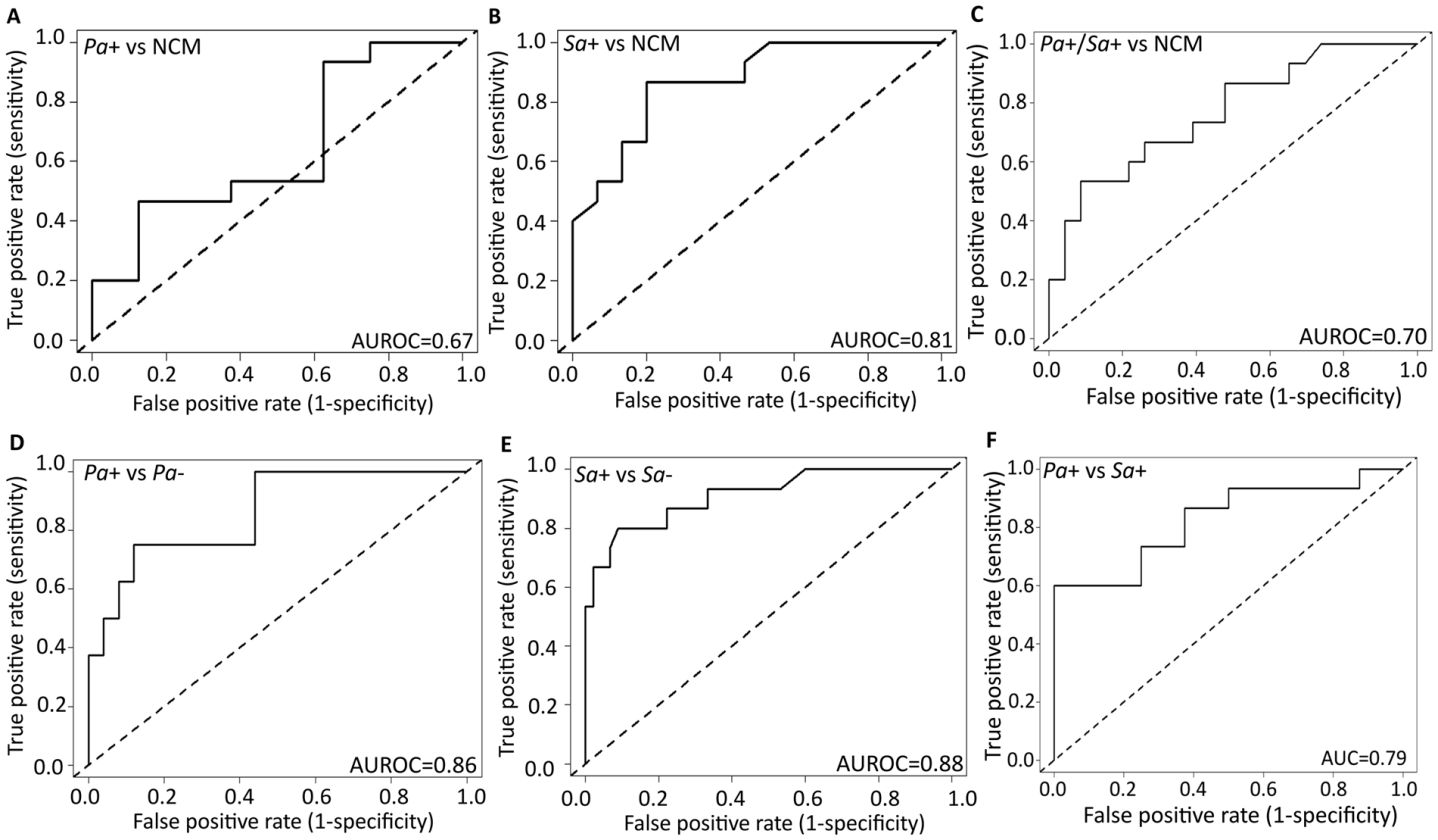
Receiver operating characteristic (ROC) curves on test set samples of (**A**) *Pa*+ (n = 8) versus NCM (n = 15) using a panel of six volatile molecules, (**B**) *Sa*+ (n = 15) versus NCM (n = 15) using seven volatile molecules, (**C**) *Pa*+/*Sa*+ (n = 23) versus NCM (n = 15) using nine volatile molecules, (**D**) *Pa*+ (n = 7) versus *Pa*− (n = 53) using nine volatile molecules, (**E**) *Sa*+ (n = 15) versus *Sa*− (n = 45) using eight volatile molecules and (**F**) *Pa*+ (n  = 8) versus *Sa*+ (n  = 15) using 11 volatile molecules. (Dotted line indicates random classification).

Starting from a basis of 805 peaks, the RF classification algorithm^30^ was used to identify suites of discriminatory volatile molecules for all models. Each model was validated using a test set, and sensitivity, specificity, PPV, NPV and AUROC values were calculated (Table 2). First, we evaluated the diagnostic accuracy of volatile molecules in discriminating culture-positive from culture-negative samples. Using a set of six, seven, and nine discriminatory volatile molecules selected from training sets for *Pa*+ versus NCM, *Sa*+ versus NCM, and *Pa*+/*Sa*+ versus NCM, respectively, AUROC values of 0.67 (95% CI 0.39–0.89), 0.81 (95% CI 0.74–0.99), and 0.70 (95% CI 0.62–0.89) were obtained on respective test set samples (Fig. 3A–E). Our *Pa*+/*Sa*+ versus NCM model had a sensitivity of 0.79 and PPV of 0.81 for the presence of either *P. aeruginosa* or *S. aureus*. In addition, we tested models for *Pa*+ versus *Pa*−, which yielded an AUROC of 0.86 (95% CI 0.71–1.00) and NPV of 0.92 using nine volatile molecules, and models for *Sa*+ vs *Sa*−, which yielded an AUROC of 0.88 (0.79–1.00) and a PPV of 0.86 using eight volatile molecules.

**Table 2:** Model error rate, sensitivity, specificity, PPV, NPV, AUROC (95% CI) for volatile molecules from BAL fluid as diagnostic for *Pa*+ versus NCM, *Sa*+ versus NCM, *Pa*+/*Sa*+ versus NCM, *Pa*+ versus *Sa*+, *Sa*+ versus *Pa*+, *Pa*+ versus *Pa*− and *Sa*+ versus *Sa*−.

	n	Model error rate	Sensitivity	Specificity	PPV	NPV	AUROC
***Pa*+ vs NCM (n** = **53), volatile molecules used for test set = 6**							
Training set	30	0.30	0.35 (0.05–0.76)	0.70 (0.40–0.90)	0.30 (0.10–0.62)	0.72 (0.55–0.88)	0.70 (0.45–0.91)
Test set	23	0.40	0.29 (0.04–0.71)	0.63 (0.35–0.85)	0.25 (0.08–0.56)	0.66 (0.52–0.79)	0.67 (0.39–0.89)
***Sa*** **+ vs NCM (n = 73), volatile molecules used for test set = 7**							
Training set	43	0.32	0.58 (0.30–0.80)	0.60 (0.27–0.85)	0.62 (0.45–0.81)	0.51 (0.33–0.66)	0.87 (0.77–1.00)
Test set	30	0.46	0.53 (0.27–0.77)	0.54 (0.25–0.81)	0.60 (0.42–0.76)	0.45 (0.30–0.64)	0.81 (0.74–0.99)
***Pa*** **+/** ***Sa*** **+ vs NCM (n = 91), volatile molecules used for test set = 9**							
Training set	53	0.30	0.82 (0.66–0.95)	0.72 (0.39–0.90)	0.86 (0.71–0.95)	0.72 (0.46–0.89)	0.80 (0.69–0.97)
Test set	38	0.40	0.79 (0.63–0.90)	0.60 (0.33–0.82)	0.81 (0.70–0.89)	0.56 (0.38–0.72)	0.70 (0.62–0.89)
***Pa*** **+ vs** ***Sa*** **+ (n = 59), volatile molecules used for test set = 11** ^*^							
Training set	36	0.25	0.70 (0.35–0.93)	0.92 (0.63–1.00)	0.88 (0.51–0.98)	0.80 (0.61–0.91)	0.84 (0.71–0.98)
Test set	23	0.30	0.73 (0.46–0.87)	0.81 (0.60–0.94)	0.67 (0.43–0.85)	0.72 (0.59–0.82)	0.79 (0.67–0.95)
***Sa*** **+ vs** ***Pa*** **+ (n = 59), volatile molecules used for test set = 11** ^*^							
Training set	36	0.25	0.92 (0.63–1.00)	0.70 (0.35–0.93)	0.80 (0.61–0.91)	0.88 (0.51–0.98)	0.84 (0.71–0.98)
Test set	23	0.30	0.81 (0.60–0.94)	0.73 (0.46–0.87)	0.72 (0.59–0.82)	0.67 (0.43–0.85)	0.79 (0.67–0.95)
***Pa*** **+ vs** ***Pa−*** ** (n = 133), volatile molecules used for test set = 9**							
Training set	73	0.20	0.77 (0.60–0.90)	0.91 (0.48–0.94)	0.71 (0.37–0.80)	0.95 (0.91–1.00)	0.90 (0.82–1.00)
Test set	60	0.25	0.75 (0.63–1.00)	0.88 (0.48–0.90)	0.67 (0.38–0.75)	0.92 (0.88–1.00)	0.86 (0.71–1.00)
***Sa*+ vs** ***Sa*− (n** = **133), volatile molecules used for test set** = **8**							
Training set	73	0.30	0.85 (0.80–0.99)	0.56 (0.36–0.79)	0.90 (0.81–0.95)	0.73 (0.65–0.80)	0.89 (0.80–1.00)
Test set	60	0.37	0.80 (0.78–0.92)	0.52 (0.31–0.72)	0.86 (0.61–0.96)	0.70 (0.61–0.75)	0.88 (0.79–1.00)

^*^Volatile molecules are the same for both models.

**Figure 4:**
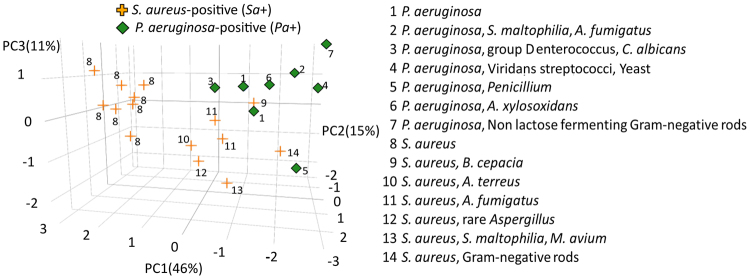
Three-dimensional principal component scores plot on test set samples of *Pa*+ (diamond, n = 8) versus *Sa*+ (plus, n = 15) with complete microbiology profiles.

To determine whether volatile molecules can differentiate between polymicrobial *P. aeruginosa*-positive and *S. aureus*-positive samples, the *Pa*+ versus *Sa*+ model was built and validated on a test group of samples using a set of 11 discriminatory volatile molecules. The model had a specificity and NPV of 0.81 and 0.72, respectively for detecting *P. aeruginosa*, sensitivity and PPV of 0.67 and 0.66, respectively for detecting *S. aureus* and an AUROC of 0.79 (95% CI 0.67–0.95) (Fig. 3F). PCA plot was used to visualize the clustering of test set samples using discriminatory volatiles for the *Pa*+ versus *Sa*+ model, with labels indicating the complete clinical microbiology profiles (Fig. 4). Samples that cultured only *S. aureus* (profile #8, n = 8) clustered together, while samples that cultured *S. aureus* with additional Gram-negative microorganisms (profiles #9, #14, n = 2), or *S. aureus* along with *Aspergillus* spp. (profiles #10, #11, #12, n = 4) clustered closer to *P. aeruginosa* samples.

In pediatric patients, *S. aureus* pre-colonization has been shown to be a risk factor for initial *P. aeruginosa* airway infection^33^. Hence, it is important to assess the utility of diagnostic biomarkers in *S. aureus* and *P. aeruginosa* co-infected samples. Using the panel of our 28 *Pa*+ biomarkers identified from all our models above, we tested whether samples that were co-infected with *P. aeruginosa* and *S. aureus* (n = 9) could be identified as *Pa*+. We added these samples in our test set of *Pa*+ versus *Pa*− analysis and achieved a sensitivity and specificity of 0.74 and 0.89, suggesting that our reported suite has the potential to rule in *P. aeruginosa* in polymicrobial samples.

Putative identifications and relative abundance of the 38 discriminatory volatile molecules from all six models were determined using mass spectrometric and chromatographic data (Table 3, Supplementary Figures S2–S7). MANOVA was used to ensure that the volatile profile was not confounded by clinical and demographic characteristics of CF patients in Table 1. None of the characteristics significantly influenced the models (Supplementary Table S1). Six discriminatory volatile molecules had been previously reported and were given putative names; 2-butanone, 2-methyl-2-butanol, 1-butanol, ethyl acetate, 2-butanol and 3-methyl-2-butanone. Two out of the six volatile molecules were associated with metabolic pathways in the KEGG database; 2-butanone and 1-butanol (Table 4).

**Table 3:** Putative identification of 38 discriminatory volatile molecules for all six models.

Peak #	Putative identification	Putative molecular formula	*Pa*+ vs NCM	*Sa*+ vs NCM	*Pa*+/*Sa*+ vs NCM	*Pa*+ *vs Sa*+	*Pa*+ *vs Pa−*	*Sa*+ *vs Sa*−	CAS#	RI
1	Unknown	Unknown	—	—	↑	—	—	—	—	—
2	Ketone	C_6_H_12_O_2_	—	—	—	↓	—	—	—	—
3	**2-butanone**	**C**_**4**_ **H**_**8**_ **O**	↑**	↑**	↑*	**↓**	↑*	—	**78-93-3**	**634**
4	Ether	C_4_H_6_O	—	—	—	↑	—	—	—	635
5	**2-methyl-2-butanol**	**C**_**5**_ **H**_**12**_ **O**	**—**	**—**	**—**	**↑**	**—**	**—**	**75-85-4**	**686**
6	**1-butanol**	**C**_**4**_ **H**_**10**_ **O**	**—**	**—**	**—**	**↓**	**—**	**—**	**71-36-3**	**717**
7	Aromatic	C_6_H_8_O	↓	↑	↑	—	—	↑	—	734
8	Ether	C_6_H_12_O_2_	—	—	—	↑	—	—	—	744
9	Ketone	C_6_H_12_O	—	—	—	↑	—	—	—	743
10	Sulfur Containing	C_20_H_42_O_2_S	—	—	—	↑	—	—	—	788
11	Hydrocarbon	C_15_H_30_	↑*	—	↑	—	—	—	—	795
12	Ketone	C_9_H_16_O_3_	—	—	↑	—	—	—	—	826
13	Alcohol	C_11_H_22_O_2_	—	—	↑	—	—	—	—	841
14	Ester	C_5_H_8_O_4_	—	↑*	—	↓	—	↑	—	—
15	Hydrocarbon	C_10_H_20_	↑*	—	↑*	—	—	—	—	—
16	Nitrogen Containing	C_12_H_25_NO_2_	—	—	—	↓	—	—	—	—
17	Carboxylic Acid	C_4_H_6_O_4_	—	—	↑*	—	—	—	—	—
18	Ketone	C_12_H_14_O	—	—	↓	—	—	—	—	—
19	Alcohol	C_4_H_10_O_3_	—	—	—	↑	—	—	—	—
20	Hydrocarbon	C_4_H_8_	↑	—	—	—	↑**	—	—	—
21	Ester	C_4_H_8_O_2_	↑	—	—	—	—	—	—	—
22	Hydrocarbon	C_8_H_18_	—	↑	—	—	—	—	—	734
23	Alcohol	C_5_H_12_O	—	↑**	—	—	—	—	—	768
24	Alcohol	C_16_H_14_O	—	↓**	—	—	—	—	—	789
25	Hydrocarbon	C_9_H_20_	—	↑**	—	—	—	—	—	—
26	**Ethyl acetate**	**C**_**4**_ **H**_**8**_ **O**_**2**_	—	—	—	—	↓**	—	**141-78-6**	**638**
27	**2-butanol**	**C**_**4**_ **H**_**10**_ **O**	—	—	—	—	↓*	—	**78-92-2**	**686**
28	**3-methyl-2-butanone**	**C**_**5**_ **H**_**10**_ **O**	—	—	—	—	↑*	—	**563-80-4**	**700**
29	Ester	C_6_H_12_O_2_	—	—	—	—	↑*	—	—	784
30	Hydrocarbon	C_4_H_10_	—	—	—	—	↑*	—	—	—
31	Aromatic	C_8_H_10_	—	—	—	—	↑*	—	—	—
32	Nitrogen containing	C_10_H_15_N	—	—	—	—	↓**	—	—	—
33	Ether	C_4_H_6_O	—	—	—	—	—	↑	—	—
34	Hydrocarbon	C_7_H_16_	—	—	—	—	—	↑	—	—
35	Ketone	C_6_H_12_O	—	—	—	—	—	↑	—	—
36	Ketone	C_8_H_16_O	—	—	—	—	—	↑	—	—
37	Aromatic	C_14_H_22_	—	—	—	—	—	↑	—	—
38	Ketone	C_7_H_14_O	—	—	—	—	—	↑**	—	—

Volatile molecules in **bold** have been previously reported in the literature in *P. aeruginosa* and/or *S. aureus* volatile metabolomics studies. RI; experimentally determined retention-index, Up; ↑, Down; ↓, based on mean peak area. p-value <0.05*, 0.01** considered statistically significant after Benjamini-Hochberg correction.

**Table 4:** Previously reported *P. aeruginosa–* and *S. aureus–*-associated volatile molecules.

Volatile molecule	KEGG pathway ID	Pathway name	Reference		
			*Pa*	*Sa*	Both
2-butanone	00460	Cyanoamino acid metabolism	^23,34,35,49–52^	^24^	^44^
	01110	Biosynthesis of secondary metabolites			
2-methyl-2-butanol	—	—	^51^	—	—
1-butanol	00650	Butanoate metabolism	^17,18,49–51^	—	^44,53,54^
	01120	Microbial metabolism in diverse environments			
	01220	Degradation of aromatic compounds			
2-butanol	—	—	^44^	—	^50^
Ethyl acetate	—	—	^51^	—	^44,53^
3-methyl-2-butanone	—	—	^35^	—	—

## Discussion

This is the first study to examine the diagnostic potential of a suite of volatile molecules in identifying the presence of two critical lung pathogens for patients with cystic fibrosis, *P. aeruginosa* and *S. aureus*. The molecules identified in the headspace of BAL fluid were similar in chemical class composition to previously reported culture and exhaled breath studies^34–38^. The range of models presented for the detection of each pathogen were validated using test sets and yield a set of putative discriminatory biomarkers for translation and evaluation in future breath studies.

In non-expectorating subjects, clinicians use OP swab culture as a surrogate for BAL fluid. Jung *et al*. reported a PPV and NPV of 0.83 and 0.74 respectively, for detecting *P. aeruginosa* using OP swab culture and a PPV and NPV of 1.00 and 0.94, respectively, using sputum culture in 38 patients^13^. Seidler *et al*. reported that in expectorating adults (n = 20), OP swab and sputum cultures both had a PPV of 1.00 for detecting *P. aeruginosa* and NPV of 0.50 and 0.60, respectively^39^. In this work, 28 putative volatile biomarkers were selected across all our models for the identification of *P. aeruginosa* in BAL fluid samples yielding a PPV range of 0.25–0.81 and an NPV range of 0.56–0.92. Our *Pa*+ versus *Pa−* model generated a specificity of 0.88 and an NPV of 0.92, suggesting that volatile molecules from the headspace of BAL fluid could be useful to screen patients and ‘rule in’ *P. aeruginosa* infections. Clinical best practice, as defined by the CF Foundation Pulmonary Guidelines, is to routinely surveil CF patients for new or recurring *P. aeruginosa* infections and initiate antibiotic eradication therapy at first detection, in an effort to prevent chronic infection and preserve lung function^4^. Ruling in *P. aeruginosa* is important as presence of the bacterium in the lung is significantly correlated with lower FEV_1_% predicted, increased serum C-reactive protein, and increased neutrophil elastase in sputum^40^.

Identification of *S. aureus* is also important, as methicillin-resistant *S. aureus* (MRSA) is associated with worse survival in CF patients^5^. Seidler *et al*. reported an NPV of 1.00 for detecting *S. aureus* in OP swab and sputum cultures versus BAL fluid and a PPV of 0.41 for OP swab and 0.57 for sputum versus BAL fluid^39^. Neericnx *et al*. demonstrated that a combination of nine volatile molecules in breath were able to discriminate between CF patients infected with *S. aureus* (n = 13) and uninfected CF patients (n = 5) with a sensitivity of 1.00 and a specificity of 0.80^24^. We identified 30 volatile molecules across all of our *S. aureus* models which yielded a PPV range of 0.60–0.86 and an NPV range of 0.37–0.70, respectively. Our *Sa*+ versus *Sa−* model generated a sensitivity of 0.80 and a PPV of 0.86, indicating that volatile molecules from BAL fluid samples could be used to ‘rule out’ *S. aureus*. The determination of *S. aureus* infection via volatile molecule panels is a promising avenue of research, however, the development of reliable exhaled breath diagnostics for *S. aureus* will require the use of paired representative samples from the lower and upper airways of subjects with CF to rule out false positives, as *S. aureus* is a colonizer of the upper airways of persons with CF^41^.

Six of the 38 discriminatory volatile molecules have been previously identified as bacterially-derived in volatile metabolomics studies (Table 4). Of these, the KEGG database identified microbial pathways associated with the metabolism of 2-butanone and 1-butanol. Of interest was the release of 2-butanone as a secondary metabolite during a reaction that also results in the formation of hydrogen cyanide (HCN). Hydrogen cyanide is an extensively studied biomarker for *P. aeruginosa* identification^19–21^ and more recently also shown to be produced by *S. aureus*^42^. We do not report on HCN (27.0 amu) as we did not collect data on ions with a m/z < 30. 2-butanone has also been reported as a biomarker for *S. aureus* detection in the breath of CF patients^24^, indicating the potential translation of BAL fluid volatile biomarkers to patient breath. KEGG pathways associated with 1-butanol indicate that it is produced as a by-product of the reversible oxidation of butane and butanal, a metabolic pathway that has been reported in *P. butanavora*^43^. 1-butanol has also been reported as a putative biomarker in the breath of CF patients with *P. aeruginosa* infection^17^. In addition, production of 1-butanol via the glycolytic fermentation of pyruvate has been reported for *S. aureus*^44,45^. These findings point towards possible microbial origins of 2-butanone and 1-butanol in BAL fluid samples.

Although culture remains the gold standard for clinical diagnosis, molecular-based approaches can identify additional microbial species that were not cultured, some of which may affect clinical outcomes in CF patients^46–48^. To the best of our knowledge, no volatile metabolomics study to-date has compared culture-dependent and culture-independent techniques for the purpose of organism identification. For our *Pa*+ vs *Sa*+ model, we compared the bacterial classifications based on 16 S ribosomal RNA (rRNA) sequencing (see supplementary information for details) and culture data. Our results show that samples with concordant microbiological results (average predicted probabilities; *Pa*+ = 0.70, *Sa*+ = 0.75) showed higher predicted probabilities than samples that had discordant microbiological findings (average predicted probabilities; *Pa*+ = 0.35, *Sa*+ = 0.30) (Supplementary Figure S8). Although the predicted probability of samples with concordant microbiology did not change, indicating the model was robust, the choice of reference sample for model generation in the field of volatile metabolomics is an unexplored issue. Future studies for *P. aeruginosa* and *S. aureus* volatile biomarkers should consider incorporating 16 S rRNA or quantitative polymerase chain reaction (qPCR) in order to determine sample status for predictive modeling.

CF lung infections are polymicrobial and our data shows that suites of volatile molecules from BAL fluid produced a predictive signal. We recognize that we have not fully investigated the impact of multiple pathogens on our reported volatile biomarker panel. This was beyond the scope of the study and shall be explored as a future avenue. As a first pass to address this question, we used our 28 *Pa*+ biomarkers to test whether the samples that were co-infected with *P. aeruginosa* and *S. aureus* could be identified as *Pa*+. Our sensitivity and specificity were 0.74 and 0.89 when these samples were included in the test set of *Pa*+ versus *Pa*− model, demonstrating the utility of the volatile biomarkers to rule in *P. aeruginosa* in polymicrobial samples. In addition, we acknowledge that using the BAL technique to obtain diagnostic samples is invasive, expensive, and thus cannot be performed frequently in a clinical setting. We do not propose the use of volatile molecule suites from BAL fluid as a rapid or non-invasive diagnostic in clinical labs. Furthermore, the PPV and NPV values for our study might not be reflective of the actual prevalence of *P. aeruginosa* and *S. aureus* in all CF populations. However, the proposed suite of biomarkers in this study can now serve as a basis for designing well-powered breath studies in the CF patient population, an avenue we are currently exploring. Furthermore, we expect that complementing the culture results with culture-independent data might have the potential to identify additional, clinically relevant microbial species, some of which may affect model generation and interpretation^46–48^. We conclude that volatile molecules from BAL fluid can provide discriminatory power for ruling in *P. aeruginosa* and ruling out *S. aureus* and plan to extend this work to exhaled breath in CF patients.

## Electronic supplementary material

Supplementary information
