# Supplementary material for: Volatile molecules from bronchoalveolar lavage fluid can ‘rule-in’ Pseudomonas aeruginosa and ‘rule-out’ Staphylococcus aureus infections in cystic fibrosis patients
Source: Sci Rep. 2018 Jan 16;8:826. doi: 10.1038/s41598-017-18491-8 (PMC5770459; doi:10.1038/s41598-017-18491-8)
Supplement: Supplementary file 1 — Supplementary information [file 41598_2017_18491_MOESM1_ESM.pdf]

Volatile molecules from bronchoalveolar lavage fluid can 'rule-in' *Pseudomonas aeruginosa* and 'rule-out' *Staphylococcus aureus* infections in cystic fibrosis patients

Mavra Nasir[1], Heather D. Bean[2], Agnieszka Smolinska[3], Christiaan A. Rees[1], Edith T. Zemanick[4], Jane E. Hill*[1,5]

[1]Geisel School of Medicine, 1 Rope Ferry Road, Dartmouth College, Hanover, NH, 03755, United States.

[2]School of Life Sciences, Arizona State University, 427 East Tyler Mall, Tempe, AZ, 85287, United States.

[3]NUTRIM School of Nutrition and Translational Research in Metabolism, Department of Pharmacology and Toxicology, Maastricht University, Maastricht, The Netherlands.

[4]School of Medicine, Colorado Anschutz Medical Campus and Children's Hospital Colorado, Department of Pediatrics, 13123 E 16th Avenue, Aurora, CO, 80045, United States.

[5]Thayer School of Engineering, 14 Engineering Drive, Dartmouth College, Hanover, NH, 03755, United States.

Correspondence to Jane E. Hill, Associate Professor of Engineering, Thayer School of Engineering, 14 Engineering Drive, Hanover, NH, 03755, USA; jane.e.hill@dartmouth.edu; +1-603-646-8656

ONLINE SUPPLEMENTARY DATA

Materials and Methods

BAL fluid sample measurements by two-dimensional gas chromatography-time-of-flight mass spectrometry. Samples were randomized before analysis. Five hundred microliters of thawed BAL fluid were transferred to sterile 10 mL headspace vials containing a magnetic stir bar and sealed with a polytetrafluoroethylene/silicone screw cap (Sigma-Aldrich, St. Louis, MO). Samples were stored at 4 °C prior to analysis by comprehensive two-dimensional gas chromatography – time-of-flight mass spectrometry (GC×GC-TOFMS; Pegasus 4D, LECO Corporation, St Joseph, MI, USA). Samples were warmed to 25 °C and stirred at 250 rpm while headspace volatiles were concentrated for 60 min onto a 2 cm Divinylbenzene/Carboxen/Polydimethylsiloxane (DVB/CAR/PDMS) solid-phase microextraction (SPME) fiber (Supelco, Bellefonte, PA, USA). The GC×GC-TOFMS was equipped with a rail autosampler (MPS, Gerstel, Linthicum Heights, MD, USA) and fitted with a two-dimensional column set consisting of an Rxi-624Sil-MS (60 m × 250 μm x 1.4 μm (length x internal diameter x film thickness); Restek, Bellefonte, PA, USA) first column followed by a Stabilwax (Crossbond Carbowax polyethylene glycol; 15 m x 250 μm x 0.5 μm; Restek, Bellefonte, PA) second column. The primary oven temperature containing column 1 was ramped at 3.5 °C/min from 35 °C to 230 °C. The secondary oven containing column 2, and the quad-jet modulator (2.0 s modulation period, 0.5 s alternating hot and cold pulses), were heated in step with the primary oven with +5 °C and +25 °C offset relative to the primary oven, respectively. The helium carrier gas flow rate was 2 mL/min. A splitless injection was used with a 180 s desorption time. The inlet and transfer line temperatures were set to 250 °C. Mass spectra were acquired over the range of 30 to 500 a.m.u., with an acquisition rate of 200 spectra/s. Data acquisition and chromatographic alignment were performed using ChromaTOF software version 4.50 (LECO Corporation). For the alignment of peaks across chromatograms, maximum first and second-dimension retention time deviations were set at 6.0 s and 0.15 s, respectively. For peak identification, a S/N ratio of 20:1 was required in at least one chromatogram, and a minimum of 5:1 in all others. The resulting peaks were identified by a forward search of the NIST 2011 library, and a match score of at least 850/1000 was required for putative compound

identification. Retention indices (RI) were calculated using external alkane standards (C_6 – C_{15}). Headspace volatile molecules of a pure retention index (RI) mixture (Sigma-Aldrich, St. Louis, MO) were sampled using the 2 cm triphase SPME fiber for 10 min at 50 °C and desorbed at a 30:1 split. RIs for compounds eluting prior to hexane (C_6) or after pentadecane (C_{15}) were extrapolated.

DNA extraction for 16S rRNA sequencing and alignment was done as previously described[1]

Results

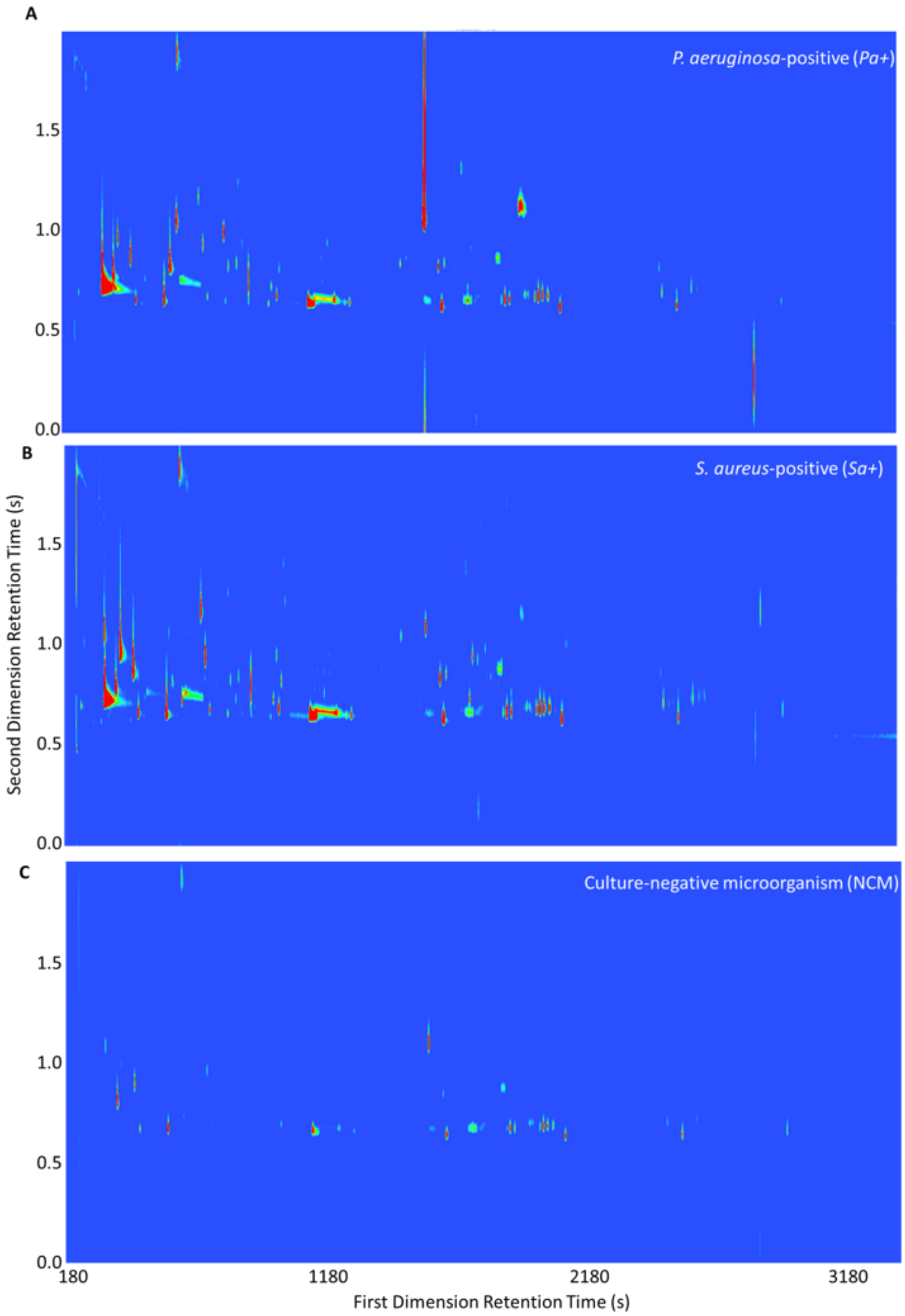

Figure S1 Representative two-dimensional gas chromatograms of BAL fluid from **(A)** *P. aeruginosa*-positive (*Pa*+), **(B)** *S. aureus*-positive (*Sa*+), and **(C)** culture-negative (or "no cultured microorganisms", NCM) groups.

For all box-whisker plots below: The lower, middle and upper lines of the box correspond to the first, second and third quartiles (the 25th, 50th and 75th percentiles). The upper whisker extends from the upper line to the largest value no further than 1.5 * IQR from the line (where IQR is the inter-quartile range). The lower whisker extends from the bottom line to the smallest value at most 1.5 * IQR of the range. Data beyond the end of the whiskers are called "outlying" points and are plotted individually. **(Peaks are arranged in order of peak number from chromatographic alignment).**

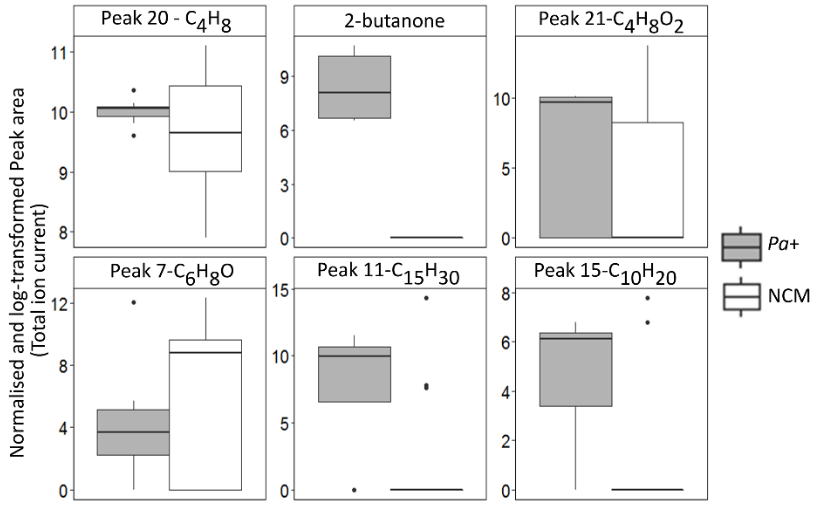

Figure S2 Box plot of six discriminatory volatile molecules used for test set samples of *Pa*+ (grey) versus NCM (white) model.

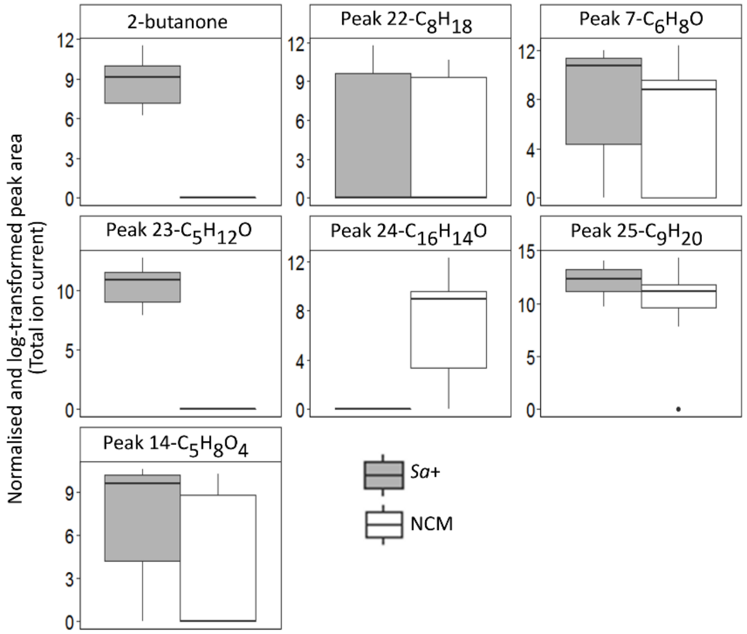

Figure S3 Box plot of seven discriminatory volatile molecules used for test set samples of *Sa*+ (grey) versus NCM (white) model.

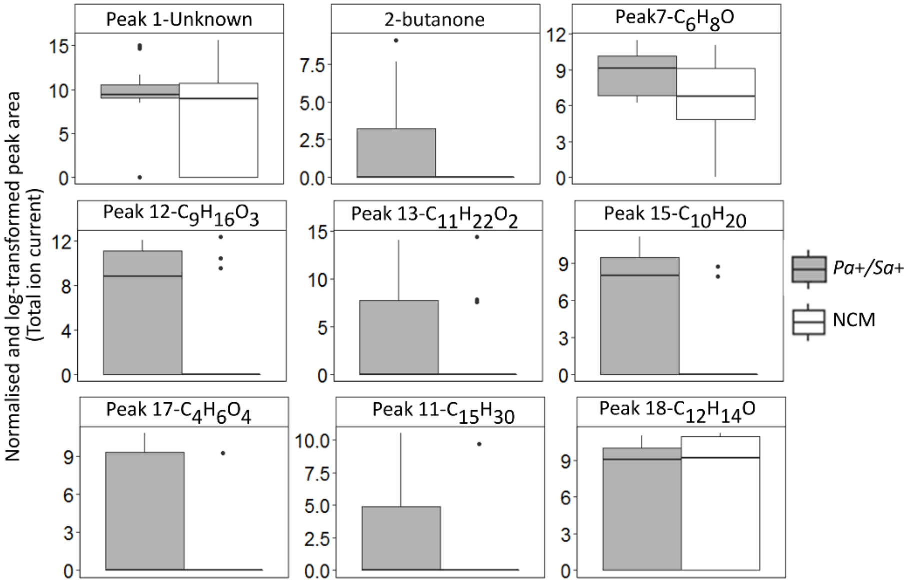

Figure S4 Box plot of nine discriminatory volatile molecules used for test set samples of *Pa*+/*Sa*+ (grey) versus NCM (white) model.

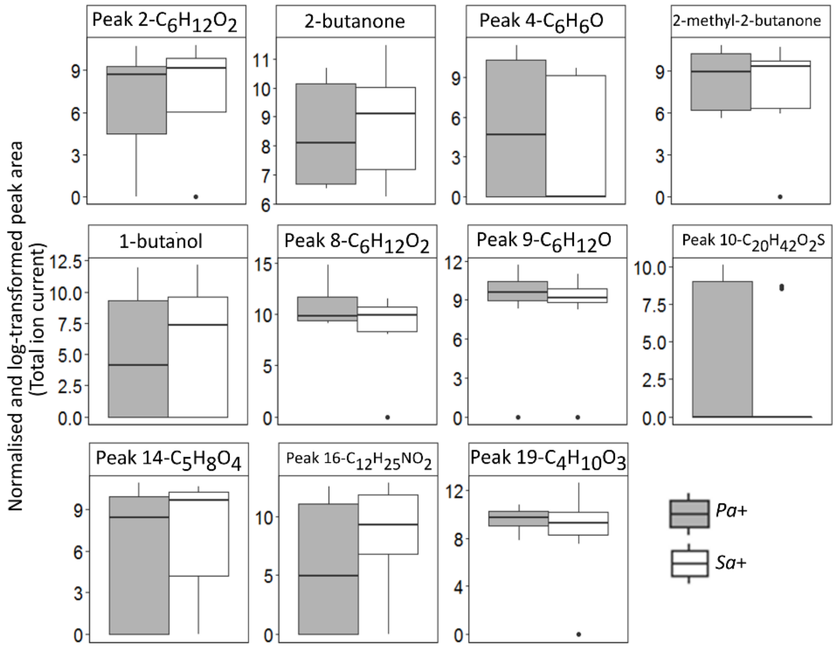

Figure S5 Box plot of 11 discriminatory volatile molecules used for test set samples of *Pa*+ (grey) versus *Sa*+ (white) model.

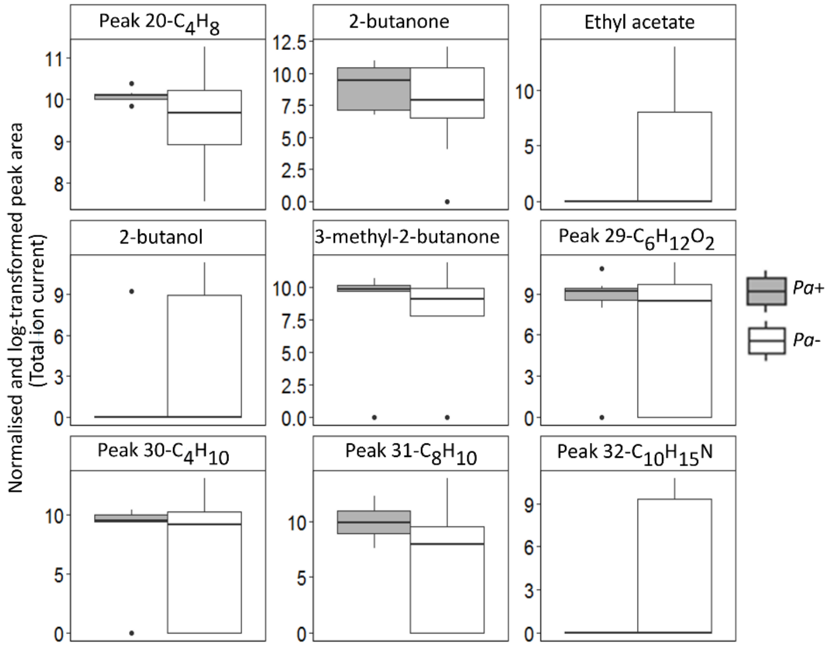

Figure S6 Box plot of nine discriminatory volatile molecules used for test set samples *Pa*+ (grey) versus *Pa*- (white) model.

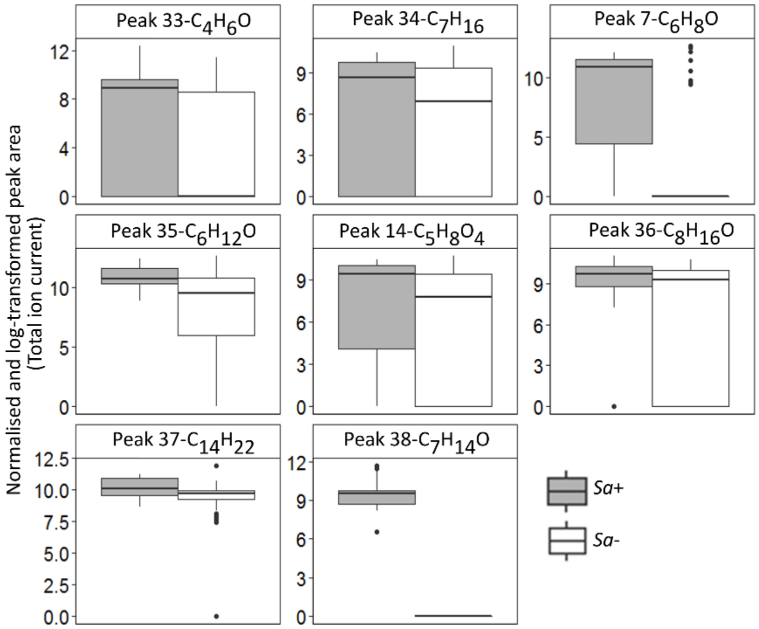

Figure S7 Box plot of eight discriminatory volatile molecules used for test set samples of *Sa*+ (grey) versus *Sa*- (white) model.

Table S1 Statistical significance of confounding factors tested by multivariate analysis of variance (MANOVA)[2] for discriminatory volatile molecules for *Pa*+ versus NCM, *Sa*+ versus NCM, *Pa+/Sa*+ versus NCM, *Pa*+ versus *Sa+*, *Pa*+ versus *Pa-*, and *Sa*+ versus *Sa*- BAL fluid samples.

	**p-values**					
**Confounders**	***Pa*+ vs NCM**	***Sa*+ vs NCM**	***Pa+/Sa*+ vs NCM**	***Pa*+ vs *Sa+***	***Pa*+ vs *Pa-***	***Sa*+ vs *Sa-***
Age	0.24	0.56	0.47	0.07	0.08	0.32
Gender	0.67	0.59	0.44	0.30	0.25	0.11
BMI	0.35	0.21	0.34	0.09	0.08	0.32
Genotype	0.45	0.36	0.33	0.44	0.17	0.15
Comorbidities						
Pancreatic insufficiency	0.47	0.54	0.35	0.67	0.30	0.27
CF-related diabetes	0.35	0.23	0.13	0.23	0.22	0.43
FEV_1 % predicted	0.43	0.49	0.65	0.50	0.37	0.55
FVC_1 % predicted	0.80	0.31	0.76	0.70	0.45	0.64

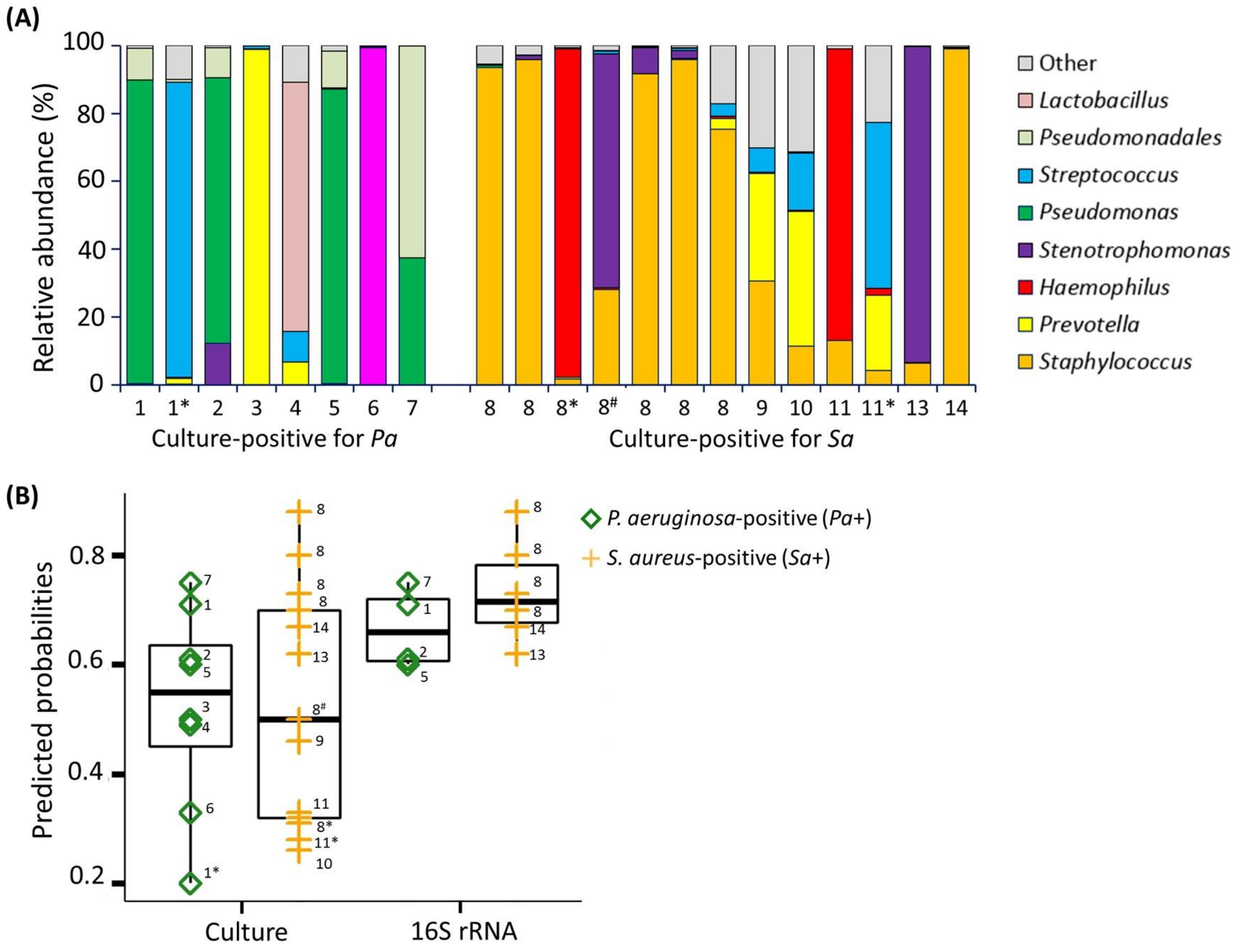

Figure S8 Comparison of *Pa*+ vs *Sa*+ model performance on test set samples based on culture versus 16S rRNA results **(A)** Barplot showing the relative abundance of genus based on 16S rRNA data. Numbers on the x-axis correspond to PCA model (Figure 4 in manuscript). **(B)** Predicted probabilities for each sample based on the culture and 16S rRNA profile. (16S rRNA data was not available for sample 12 in culture-positive *Sa* group).

REFERENCES

1 Zemanick, E. T. *et al.* Airway microbiota across age and disease spectrum in cystic fibrosis. *Eur Respir J* **50**, doi:10.1183/13993003.00832-2017 (2017).

2 Stahle, L. & Wold, S. Multivariate-Analysis of Variance (Manova). *Chemometr Intell Lab* **9**, 127-141 (1990).
